# Corticomuscular interactions during different movement periods in a multi-joint compound movement

**DOI:** 10.1038/s41598-020-61909-z

**Published:** 2020-03-19

**Authors:** Rouven Kenville, Tom Maudrich, Carmen Vidaurre, Dennis Maudrich, Arno Villringer, Vadim V. Nikulin, Patrick Ragert

**Affiliations:** 1 Institute for General Kinesiology and Exercise Science, Faculty of Sports Science, University of Leipzig, D-04109 Leipzig, Germany; 2 Max Planck Institute for Human Cognitive and Brain Sciences, Department of Neurology, D-04103 Leipzig, Germany; 3 Centre for Cognition and Decision Making, National Research University Higher School of Economics, Moscow, 101000 Russian Federation; 4 Neurophysics Group, Department of Neurology, Charité-University Medicine Berlin, Campus Benjamin Franklin, Berlin, 10117 Germany; 5 Dpt. of Statistics, Informatics and Mathematics, Public University of Navarre, Pamplona, 31006 Spain; 6 Machine Learning Group, Faculty of EE and Computer Science, TU Berlin, Berlin, 10587 Germany; 7 MindBrainBody Institute at Berlin School of Mind and Brain, Charité-Universitätsmedizin Berlin and Humboldt-Universität zu Berlin, Berlin, 10099 Germany; 8 Clinic for Cognitive Neurology, University Hospital Leipzig, D-04103 Leipzig, Germany

**Keywords:** Neuroscience, Physiology

## Abstract

While much is known about motor control during simple movements, corticomuscular communication profiles during compound movement control remain largely unexplored. Here, we aimed at examining frequency band related interactions between brain and muscles during different movement periods of a bipedal squat (BpS) task utilizing regression corticomuscular coherence (rCMC), as well as partial directed coherence (PDC) analyses. Participants performed 40 squats, divided into three successive movement periods (Eccentric (ECC), Isometric (ISO) and Concentric (CON)) in a standardized manner. EEG was recorded from 32 channels specifically-tailored to cover bilateral sensorimotor areas while bilateral EMG was recorded from four main muscles of BpS. We found both significant CMC and PDC (in beta and gamma bands) during BpS execution, where CMC was significantly elevated during ECC and CON when compared to ISO. Further, the dominant direction of information flow (DIF) was most prominent in EEG-EMG direction for CON and EMG-EEG direction for ECC. Collectively, we provide novel evidence that motor control during BpS is potentially achieved through central motor commands driven by a combination of directed inputs spanning across multiple frequency bands. These results serve as an important step toward a better understanding of brain-muscle relationships during multi joint compound movements.

## Introduction

Many muscles are involved in the execution and control of a bipedal squat (BpS)^1–3^, with Solomonow, *et al*.^4^ estimating over 200 muscles to be recruited. In contrast to simple movements, BpS, as well as compound everyday life activities, i.e. walking stairs, picking up loads or carrying loads across distance, require extensive intra- and interlimb coordination^5^, which is why BpS is an ideal, naturalistic model for compound movement control. In contrast to simple movements, voluntary control of each muscle during BpS seems unlikely, especially when considering varying requirements on acting muscles due to changes in muscle function throughout different movement periods. Movement periods, i.e. dynamic (eccentric (ECC) and concentric (CON)) and static (isometric (ISO)) contraction periods require all muscles involved to dynamically change their function throughout the movement. This is evident for example from elevated proprioception, reflected by increased muscle spindle activities during eccentric contractions compared to both isometric and concentric contractions^6,7^. Thus, BpS demands continuous, extensive central-nervous information integration while placing high physical stress on the body^1–3^, signifying that motor commands have to be flexibly deployed and adapted throughout this movement in order to enable successful execution. Frequency band related neural synchrony between brain regions and muscles, detectable through corticomuscular coherence (CMC) measurements^8,9^, potentially provides an efficient solution to the challenge of enabling dynamic information processing on a whole body level during compound movement control. Indeed, supporting evidence has been provided by studies that observed frequency and amplitude modulations of CMC in beta and gamma bands during different movement periods of simple movements, such as knee flexions^10^ and ankle flexions^11^ as well as during different stages of more compound gait cycles^12^, indicating frequency band related cortical oscillations to play a crucial role concerning flexible motor control.

During motor actions, neural oscillations are mainly observed at beta (~12–30 Hz) and gamma frequencies (~>30 Hz). The human corticomotor drive comprises an oscillatory beta component, which enables synchronization in the beta range between targeted muscles and brain areas^9,13,14^. As such, CMC between sensorimotor areas and muscles has been frequently observed in the beta range^10,15–17^ and is noticeably present during isometric movements^9,18,19^. Gamma oscillations commonly occur during movement onsets, whereby changes in oscillatory gamma activity are interlinked with varying movement properties^20,21^. Functionally, beta-range CMC has been associated with fine motor control^16,22^, motor preparation^23^ and sensorimotor integration^24^, while gamma-range CMC is assumed to reflect mechanisms underlying integration of task related cortical components during sensorimotor tasks^23,25^, proprioceptive feedback^21,26^, as well as visuomotor paradigms^27,28^. Still, these results do not imply directionality between cortex and muscle activity, as CMC is undirected. Therefore, additional partial directed coherence (PDC) analyses are essential in order to examine directionality of communication between cortex and muscles by quantifying direction of information flow (DIF)^29,30^. PDC has been used for MEG^31^, EEG^32,33^ and EMG^34^ recordings. Previous evidence on directionality between EEG and EMG recordings suggests patterns of directionality as well as band-specific dominant directions of information flow during motor tasks^32,35^. For example, it could be shown that beta CMC has strongest coupling for EEG-EMG direction during various upper extremity motor tasks^32^. EEG-EMG connectivity was also significantly elevated compared to EMG-EEG connectivity in gait^35^.

All evidence can be summarized as follows. First, previous research regarding neuromuscular interactions between brain areas and musculature has focused on simple movements with no studies examining whole body movements such as BpS. Second, when examining interactions between cortex and muscles, it is important to consider differential muscle functions, which change depending on movement periods, or rather the contraction type required during different movement periods. Third, coherence between EEG and EMG recordings has been extensively used to quantify neuromuscular communication, with beta and gamma frequencies being the most prominently observed frequency bands at which EEG and EMG recordings are coherent during motor tasks. Lastly, although CMC analyses enable an assessment of dynamic neuromuscular communication, additional measures of connectivity such as PDC are necessary to analyze directions of communication.

Based on this, we hypothesized to observe both beta and gamma CMC during BpS, with CMC modulations in dependency of movement periods and muscles, as previously observed^10,12,19^. Further, as beta CMC magnitudes have been observed to be elevated during ISO^19^, we hypothesized beta CMC magnitudes to be greatest during ISO periods, as well as gamma CMC magnitudes to be greatest during ECC, reflecting increased proprioception, which has been linked to increased muscle spindle activity during ECC^6,7^ as well as gamma CMC during movements^21,26^. According to prior evidence, we further hypothesized beta CMC to primarily reflect corticomotor drive and therefore ${{DIF}}_{{EEG}-{EMG}}$ to be most prominent for beta CMC, while gamma CMC likely mirrors integration of afferent sensorimotor information and DIF to therefore be ${{DIF}}_{{EMG}-{EEG}}$.

## Results

**Figure 1:**
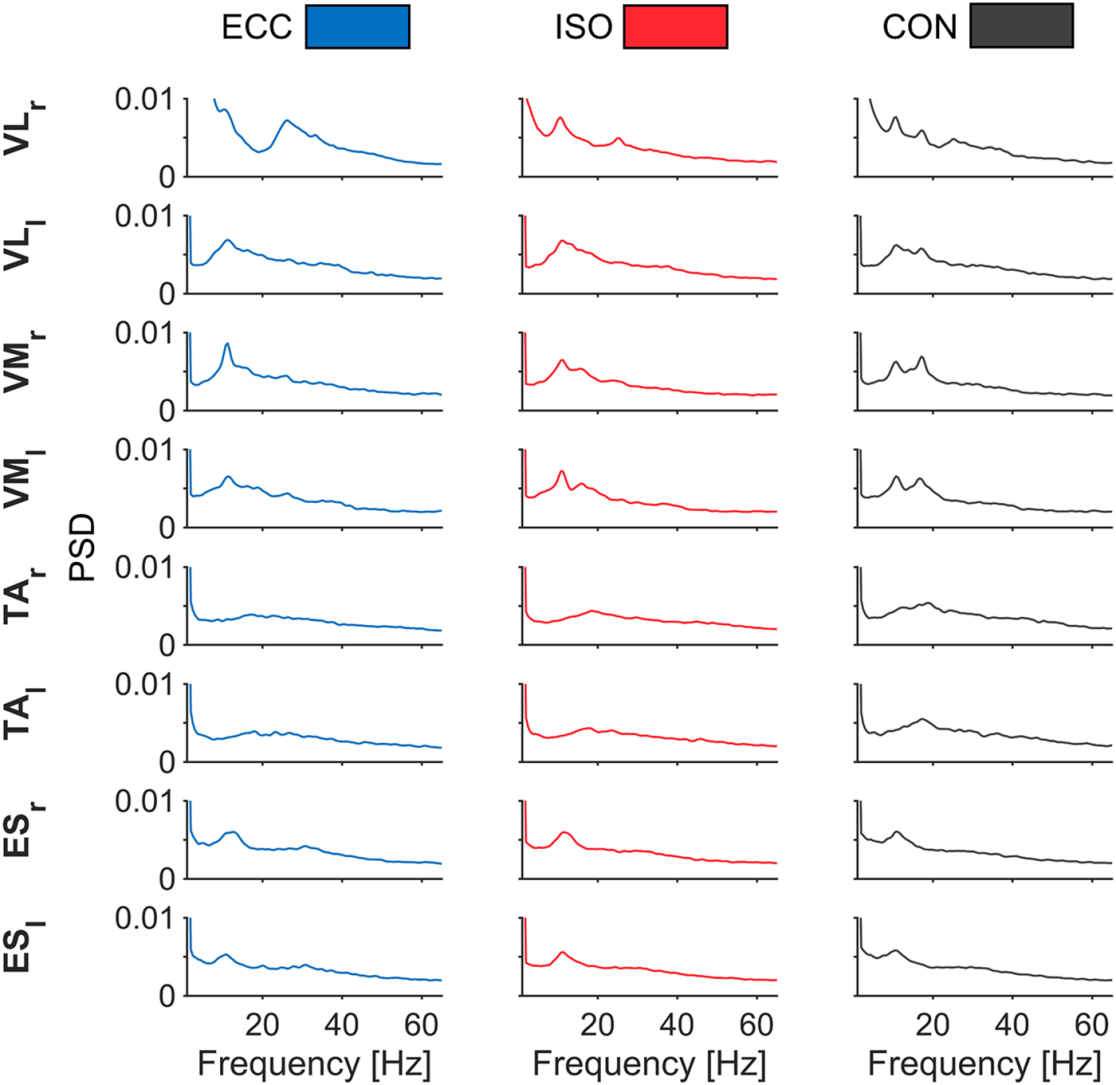
Normalized PSD of rectified EMG per muscle and period. PSD’s of rectified EMG are depicted for all muscles during each movement period. Power spectra were averaged across muscles, epochs, and participants and normalized to total power. Each column illustrates different movement periods: ECC (blue), ISO (red) and CON (gray). Each row highlights different muscles, with respective labels next to each row. Muscle names are as follows: *M. vastus lateralis* (VLr & VLl), *M. vastus medialis* (VMr & VMl), *M. tibialis anterior* (TAr & TAl), *M. erector spinae* (ESr, ESl).

Initially, we inspected normalized power spectral densities (PSD) of rectified EMG to assess spectral contents (Fig. 1). PSD’s revealed a broad spectrum with two peaks around ~10 and ~20 Hz for VL and VM, although peaks ~20 Hz were most pronounced for CON. For TA and ES, a similar spectrum yielded one peak at ~20 Hz and ~10 Hz, respectively. Another broad spectral elevation was evident in VM and VL between ~30 and ~40 Hz.

We observed CMC magnitudes between 0.013–0.228 across all participants, muscles and periods (please see Table 1 for an overview regarding CMC magnitudes).

**Table 1:** Descriptive results of CMC range (n = 11). Please note that the table is organized into three sections representing all three movement periods.

ECCENTRIC (ECC)	VLr	VLl	VMr	VMl	TAr	TAl	ESr	ESl
Mean	0.052	0.057	0.050	0.056	0.050	0.055	0.084	0.099
Std. Deviation	0.016	0.022	0.014	0.022	0.019	0.015	0.044	0.052
Minimum	0.017	0.022	0.013	0.020	0.014	0.016	0.039	0.043
Maximum	0.088	0.099	0.069	0.098	0.085	0.073	0.161	0.212
**ISOMETRIC (ISO)**								
Mean	0.057	0.063	0.062	0.067	0.061	0.065	0.075	0.073
Std. Deviation	0.027	0.046	0.042	0.054	0.024	0.028	0.042	0.035
Minimum	0.019	0.026	0.024	0.026	0.025	0.026	0.032	0.028
Maximum	0.131	0.206	0.192	0.228	0.104	0.110	0.181	0.144
**CONCENTRIC (CON)**								
Mean	0.048	0.052	0.053	0.048	0.056	0.055	0.081	0.079
Std. Deviation	0.012	0.018	0.015	0.017	0.018	0.021	0.041	0.041
Minimum	0.017	0.016	0.016	0.014	0.016	0.022	0.042	0.036
Maximum	0.061	0.091	0.073	0.075	0.090	0.105	0.177	0.180

Supplementary section.

**Figure 2:**
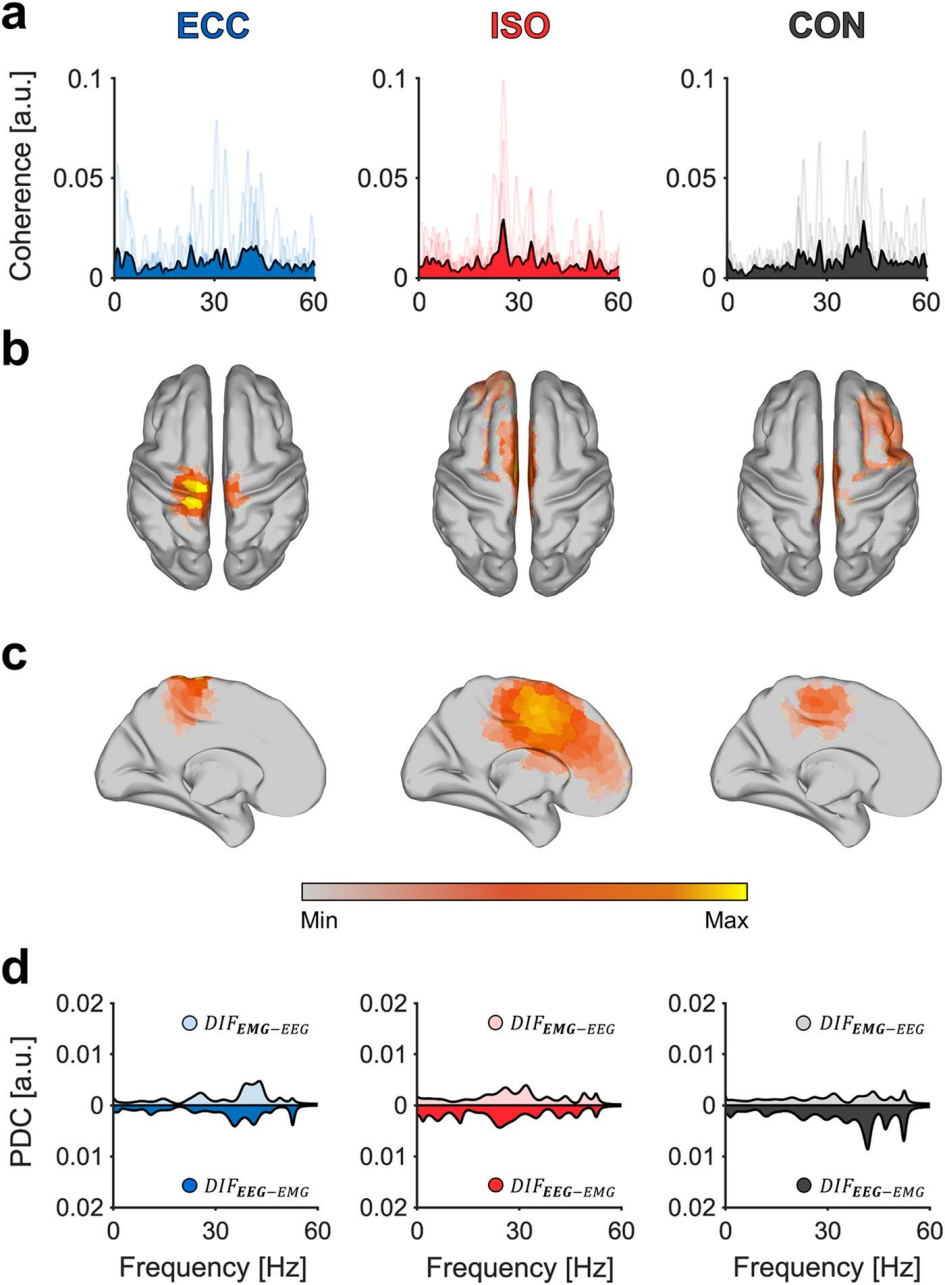
Exemplary spectral maps of CMC, PDC and source localization for TAr. Results are organized in columns representing all three movement periods during BpS. Grand-averaged **(A)** CMC spectra with individual CMC spectra indicated through transparent lines. Grand-averaged source-localization results across all movement periods displayed in **(B)** dorsal view and **(C)** mid-sagittal view and **(D)** PDC spectra during BpS execution for all movement periods. Here, PDC in EEG-EMG direction is indicated through solid areas, whereas EMG-EEG direction is indicated through transparent areas of the same color. We used the MATLAB toolbox METH by Guido Nolte (https://www.uke.de/english/departments-institutes/institutes/neurophysiology-and-pathophysiology/research/research-groups/index.html) to illustrate source localization results in sections **(B)** and **(C)**.

Figure 2 shows corticomuscular interactions between brain regions and TAr. Regarding CMC spectra of TAr, it is apparent that while individual spectral peaks vary, they accumulate within beta and gamma frequency bands (Fig. 2A). This is valid throughout all muscles, as spectral peaks rarely were identical between participants (for a complete overview regarding individual, as well as grand-averaged CMC spectra of all muscles and periods, please see Fig. S1 in supplementary section). Corresponding grand-averaged source-localization results show highest activations in contralateral M1 leg area, whereby the extent of activation varies between movement periods (Fig. 2B,C). Grand-averaged PDC spectra mirror corresponding CMC spectra as grand-averaged spectral peaks of CMC translate to those of PDC spectra. While PDC values are similar for both DIF during ISO, they are more pronounced in ${{DIF}}_{{EMG}-{EEG}}$ during ECC and ${{DIF}}_{{EEG}-{EMG}}$ during CON.

### Corticomuscular coherence during BpS (CMC)

Coherence was estimated in beta (12–30 Hz), gamma (30–44 Hz) and high gamma (44–60 Hz) frequency bands, across all muscles (8) and periods (3), resulting in 24 variables of interest for each frequency band.

**Figure 3:**
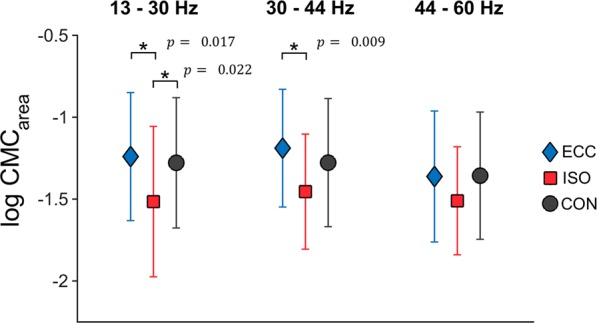
Averaged ${{\boldsymbol{CMC}}}_{{\boldsymbol{area}}}$. Averaged log-transformed ${{\boldsymbol{CMC}}}_{{\boldsymbol{area}}}$ are illustrated per movement period and frequency band across all muscles. Asterisks indicate significant differences between ${{\boldsymbol{CMC}}}_{{\boldsymbol{area}}}$ of different movement periods. Respective p values are reported next to each asterisk. Here, blue diamonds indicate ${{\boldsymbol{CMC}}}_{{\boldsymbol{area}}}$ for ECC, whereas red squares indicate ${{\boldsymbol{CMC}}}_{{\boldsymbol{area}}}$ for ISO and black circles represent ${{\boldsymbol{CMC}}}_{{\boldsymbol{area}}}$ for CON.

A two-way rmANOVA (factors: MUSCLE and PERIOD) was performed with log-transformed ${{CMC}}_{{area}}$ for each frequency band of interest. Please see Fig. 3 for an overview regarding differences in ${{CMC}}_{{area}}$ between movement periods.

For beta CMC, we did not find a significant interaction between MUSCLE*PERIOD. However, we found a significant main effect for PERIOD (F(2,20) = 9.070, p = 0.002, ${\eta }_{p}^{2}$ = 0.476) with post-hoc Bonferroni tests revealing ${{CMC}}_{{area}}$ to be higher for ECC vs. ISO (MD = 0.261, SE = 0.074, ${p}_{{bonf}.}$ = 0.017), as well as lower for ISO vs. CON (MD = −0.238, SE = 0.071, ${p}_{{bonf}.}$ = 0.022). No significant difference was found between CON vs. ECC.

For gamma CMC, we were not able to find a significant interaction between MUSCLE*PERIOD. However, we found a significant main effect for MUSCLE (F(7,70) = 2.571, p = 0.020, ${\eta }_{p}^{2}$ = 0.205); post-hoc Bonferroni tests failed to reach significance. We additionally found a significant main effect for PERIOD (F(2,20) = 8.403, p = 0.002, ${\eta }_{p}^{2}$ = 0.457) with post-hoc Bonferroni tests revealing coherence to be higher for ECC vs. ISO (MD = 0.312, SE = 0.080, ${p}_{{bonf}.}$ = 0.009).

No significant effects regarding MUSCLE (F(7,70) = 1.045, p = 0.408, ${\eta }_{p}^{2}$ = 0.095) or PERIOD (F(2,20) = 2.348, p = 0.121, ${\eta }_{p}^{2}$ = 0.190) were observed for high gamma CMC.

### Source-Localization of EEG activity (eLORETA)

Grand-averaged source-localization performed with eLORETA revealed greatest activations in contralateral (with respect to the analyzed muscle) and bilateral sensorimotor areas, with peak activities in contralateral precentral gyri and superior frontal gyri, corresponding to M1 and supplementary motor area (SMA) (please see Fig. 2B,C for exemplary grand-averaged source-localization results of TAr). Most pronounced somatotopy was visible across muscles for ISO and CON with sources being more spread out for ECC. Although sources for most muscles were located centrally in precentral gyri, in some participants, strongest activations were found in superior frontal gyri, corresponding to SMA and premotor cortex (PMC).

### Partial directed coherence during BpS (PDC)

Partial directed coherence was estimated in beta (12–30 Hz), gamma (30–44 Hz) and high gamma (44–60 Hz) frequency bands, across all muscles (8), periods (3) and for two DIF (1) ${{DIF}}_{{EEG}-{EMG}}$ and (2) ${{DIF}}_{{EMG}-{EEG}}$ (2), making up 48 variables of interest for each frequency band.

**Figure 4:**
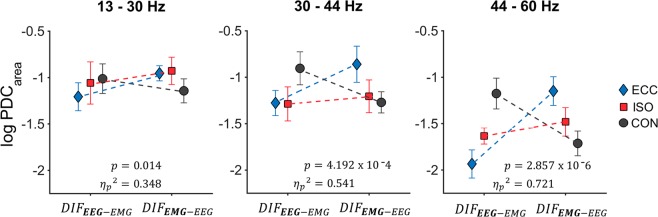
Averaged ${{PDC}}_{{area}}$ between all movement periods. Averaged log-transformed ${{PDC}}_{{area}}$ are illustrated per movement period and frequency band. Significant interactions are reported within each graph. Here, blue diamonds indicate ${{PDC}}_{{area}}$ for ECC, whereas red squares indicate ${{PDC}}_{{area}}$ for ISO and black circles represent ${{PDC}}_{{area}}$ for CON. Please note, that the x-axis represents all values for one of two possible DIF, 1) ${{DIF}}_{{EEG}-{EMG}}$ and 2) ${{DIF}}_{{EMG}-{EEG}}$.

In order to analyze directionality of obtained PDC, a three-way rmANOVA (factors: MUSCLE, PERIOD and DIRECTION) was performed between log-transformed ${{PDC}}_{{area}}$ for each frequency band of interest. Please see Fig. 4 for an overview regarding differences in DIF of ${{PDC}}_{{area}}$ between movement periods.

For beta PDC, we found a significant interaction between PERIOD*DIRECTION (F(2,20) = 5.340, p = 0.014, ${\eta }_{p}^{2}$ = 0.348). Simple effect tests showed this interaction to be present in ECC (p = 0.014, ${\alpha }_{{corr}}$ = 0.017) period. We additionally found a significant main effect for MUSCLE (F(7,70) = 2.704, p = 0.015, ${\eta }_{p}^{2}$ = 0.213), although post-hoc Bonferroni tests failed to reach significance.

Regarding gamma PDC, we found a significant interaction between PERIOD*DIRECTION (F(2,20) = 11.765, p = 4.192 × 10^−4^, ${\eta }_{p}^{2}$ = 0.541). Simple effect tests showed this interaction to be present for ECC (p = 0.016, ${\alpha }_{{corr}}$ = 0.017) and CON (p = 0.002, ${\alpha }_{{corr}}$ = 0.017) periods. We additionally found a significant interaction for MUSCLE*PERIOD*DIRECTION (F(14,140) = 2.079, p = 0.016, ${\eta }_{p}^{2}$ = 0.172). Simple effect tests showed the interaction to be present in *VLl*-ECC (p = 0.014, ${\alpha }_{{corr}}$ = 0.017), as well as *TAr*-CON (p = 0.004, ${\alpha }_{{corr}}$ = 0.017), *TAl*-CON (p = 0.008, ${\alpha }_{{corr}}$ = 0.017) and *ESl*-CON (p = 0.013, ${\alpha }_{{corr}}$ = 0.017).

For high gamma PDC, we were able to find a significant interaction between PERIOD*DIRECTION (F(2,20) = 25.843, p = 2.857 × 10^−6^, ${\eta }_{p}^{2}$ = 0.721). Simple effect tests showed this interaction to be present in ECC (p = 3.724 × 10^−13^, ${\alpha }_{{corr}}$ = 0.017) and CON (1.722 × 10^−7^, ${\alpha }_{{corr}}$ = 0.017) periods.

## Discussion

The aim of this study was to examine frequency band related interactions between cortex and muscles during different movement periods of BpS. Our results provide novel evidence that motor control during BpS, serving as a model for compound movements, is in part achieved through frequency band related neural oscillations. As hypothesized, we observed CMC in beta and gamma ranges across multiple muscles involved during BpS. CMC was altered throughout different movement periods during BpS, as ${{CMC}}_{{area}}$ was greatest during dynamic movement periods (ECC and CON), yet independent of muscles. Contrary to our hypothesis, beta CMC magnitudes were not highest during CON but during ECC. On the other hand, gamma CMC magnitudes were highest during ECC as expected. PDC analyses revealed DIF between EEG and EMG to be modulated across movement periods. As hypothesized, DIF differed between dynamic movement periods ECC (${{DIF}}_{{EMG}-{EEG}}$) and CON (${{DIF}}_{{EEG}-{EMG}}$). Lastly, modeled sources, corresponding to optimized CMC patterns, were best modeled in contralateral, as well as bilateral motor cortical areas M1, SMA and PMC.

CMC has mostly been described at beta band^22,25,36,37^ and gamma band frequencies^9,10,37–39^ for upper and lower limb isolated movements. We confirm and extend these findings, as we observed beta and gamma CMC across all muscles assessed during BpS performance, although we found no significant difference in ${{CMC}}_{{area}}$ between muscles. Conversely, numerous studies show frequency and amplitude differences in CMC between muscles^40–42^. This is possibly due to the fact, that these studies tested muscle functions and therefore estimated CMC during isolated movements, whereas all muscles involved and examined during BpS likely share a common executive blueprint and act collectively to achieve the BpS. There is evidence to support this assumption, for example, Yoshida, *et al*.^43^ examined CMC during cyclical ankle movements and were able to observe beta range CMC in both m. tibialis anterior and m. gastrocnemius, serving as agonist and antagonist. Additionally, CMC between agonist-antagonist muscle pairs was also present in the beta range for elbow flexions in healthy adults^44^. Therefore, it is conceivable that frequency band stable CMC between different muscles and brain regions represents an efficient mechanism that enables multi-muscle control during compound movements.

CMC magnitudes during this study varied across all participants (please see Table 1 for an overview of CMC magnitudes). Many factors potentially influence this, for example, it could be shown that the tendency of neural populations to synchronously discharge reveals inter-individual differences^45^. Recent evidence suggests a positive correlation between CMC magnitudes and the amount of beta oscillations in both input signals thereby supporting this assumption^46^. As CMC is not strictly of efferent origin^47^, it has been proposed that differences in the density of somatosensory receptors potentially play a part in inter-individual CMC magnitude variance^46^, although this remains to be thoroughly examined. Lastly, CMC variance might reflect differences in the way EEG recordings capture individual cortical sources. Quality and therefore the success of EEG recordings are known to depend on orientations of corticospinal neurons relative to EEG electrodes, depth of those neurons relative to individual scalps, as well as skull and scalp properties such as thickness^48,49^. Uncovering the driving factors behind these differences in individual CMC magnitudes seems crucial to gain an understanding of neurophysiological mechanisms underlying CMC.

From a mechanistical point of view, gamma band oscillations are thought to reflect network integration, i.e. the capacity of anatomically linked areas to become interconnected and exchange information on a cortical level,^10,37,50,51^, whereas beta band oscillations are mainly associated with corticomotor drive^9,23,52^. In more detail, distinctions regarding efferent and afferent information flow gain relevance when functionally distinguishing gamma from beta band CMC. This is not to say that both oscillatory components are exclusively locked to either afferent or efferent information flow. Rather, although there is no consensus regarding their neural origins, previous research seemingly implies an executive, initiative role for beta oscillations during motor actions, whereas a more sensory integrative role is ascribed to gamma oscillations. Still, some results propose a more complex origin of gamma activity beyond proprioceptive feedback^53^. In sum, both beta and gamma frequencies appear to be functionally divergent during motor task performance. Our findings extend this narrative, as CMC and PDC analyses reveal differential patterns regarding movement period related communication (CMC) and information flow (PDC) between beta and gamma frequency bands. Highest ${{CMC}}_{{area}}$ were observed during dynamic movement periods (ECC and CON) when compared to ISO with significant differences between movement periods in beta and gamma ranges. Additionally, we found significant interactions between movement periods and DIF for all tested frequency bands. In line with our hypotheses, ${{PDC}}_{{area}}$ during CON was highest in ${{DIF}}_{{EEG}-{EMG}}$, ${{PDC}}_{{area}}$ during ISO remained relatively stable across directions and ${{PDC}}_{{area}}$ during ECC was highest in ${{DIF}}_{{EMG}-{EEG}}$. This effect intensified from beta to gamma ranges, as indicated by an increase in effect sizes of movement period - DIF interactions across frequency band related ${{PDC}}_{{area}}$.

Physiologically, increased ${{DIF}}_{{EMG}-{EEG}}$ during ECC appears reasonable, as it is established that eccentric contractions prompt differential effects on muscle proprioception, e.g. increased afferent input through muscle spindles, compared to isometric and concentric contractions^54^. Eccentric contractions cause greater afferent transmission through elevated muscle spindle activity^6^, leading to increasing integrative demands on central nervous processing. Additionally, previous studies mostly observed gamma band CMC during movements in need of continuous peripheral updates, either because of great strength requirements^25,39^ or the overall novelty of the movement^10,37^, hinting at a connection between novel motor control scenarios, increased afferent information flow and gamma range CMC. Interestingly, Volgushev *et al*.^55^ argued that individual cortical neurons demonstrate more periods of enhanced excitability at gamma frequencies when compared to the beta range, which might lead to a higher potential of coherent networks to integrate task-relevant information since temporal intervals between successive time windows of increased excitability in postsynaptic neurons are decreased compared to those during beta oscillations^37^. This may serve as an explanation as to why the observed PDC results are more pronounced in gamma bands when compared to the beta range, although more evidence is needed to strengthen this assumption. Still, the exact mechanism of gamma band CMC remains to be fully understood, while current research work is focusing on uncovering its neurophysiological origin^56,57^.

Conversely, ${{PDC}}_{{area}}$ during CON was highest in ${{DIF}}_{{EEG}-{EMG}}$. It is known that, compared to eccentric movements, generated force is smaller while EMG activity is higher for concentric movements^58^, which has been attributed to differences in motoneuron excitability at supraspinal and spinal levels^59,60^. Additionally, functional magnetic resonance imaging (fMRI) analyses could show that the extent of activated motor control networks was significantly lower during concentric movements when compared to eccentric movements^61,62^, whereas functional connectivity was actually increased during concentric compared to eccentric movements^63^. Therefore, it seems likely that concentric movements are more efficiently processed when compared to eccentric movements. This is possibly due to the fact that most concentric movements are executed against gravity, whereas during most eccentric movements, gravity actually potentiates the load on acting muscles. Additionally, the majority of everyday life movements are concentric, resulting in elevated potential for motor learning to occur and people experiencing better control during concentric movements^63^. Such adaptations potentially explain ${{DIF}}_{{EEG}-{EMG}}$ in beta bands during CON, as less integrative information, reflected by gamma CMC and PDC, is required in order to execute CON contractions compared to ECC. It is worth noting that the execution speed of BpS as performed during this study (15 seconds per repetition) is slower compared to its common execution speed, especially during dynamic squat assessments, where squats are performed rapidly and movement periods are more overlapped^64^. In comparison to BpS, everyday life movements are usually performed more fluently, limiting a direct transfer of our results to such movements. Nevertheless, we think it is necessary to standardize BpS in order to draw precise mechanistic conclusions. Future studies will be able to reduce the difference in performance of standardized and naturalistic movements by increasing speed and fluidity of compound movements and evidence from both standardized and naturalistic movements will aid in acquiring further knowledge regarding compound movement control.

In general, modeled sources of obtained CMC patterns were most prominent in motor control related areas such as PMC, SMA, and M1 for ECC, ISO, and CON, while sporadically covering parietal regions during ECC. Although previous studies have reported somatotopy for CMC results^8,51,65,66^, we did not observe lateralized source patterns for unilateral muscles exclusively. For some participants and muscles, we observed bilateral activation patterns, predominantly in M1, as well as SMA. This is in line with previous results reporting bilateral activation patterns of unilateral muscles during bilateral tasks and most likely reflects control of bimanual coordination^67^. In some instances, modeled sources were broadest during eccentric periods for BpS, seemingly reflecting a larger network of involved cortical areas during ECC. This finding is intriguing regarding previous TMS studies^58^, as well as the above-mentioned fMRI studies^61,62^ indicating more extensive cortical networks for eccentric contractions when compared to concentric contractions. Still, more evidence is needed for these assumptions to gain validity. Finally, due to the custom electrode setup employed within the present study, source localization could potentially be confounded. To enable correct inverse-modeling, independent of the individual EEG configuration, we simulated EEG sources in visual cortex areas paired with an EMG source. We included background noise during the generation of both EEG and EMG signals. We simulated EEG signals 31 channels and subsequently fitted to the external layer of the standard Montreal Neurological Institute (MNI) head. The head model we used, was premised on a three-compartment realistic volume conductor and has been previously utilized to calculate EEG forward solutions^68^. We band-pass filtered independent white noise in the 18–22 Hz frequency range to generate EEG oscillations. Coherent EMG activities were modeled as time-shifted versions of matching cortical oscillations. 500 uncorrelated dipoles with random orientation and distribution on the cortex were used to generate background EEG noise. These noise sources had 1/f type spectra. Background EMG noise was generated by using random Gaussian noise mixed with the signal source. The simulated data was 150-s long and sampled at 1000 Hz. The SNR of the CMC sources was 0.5 for EEG and EMG. We applied rCMC to the simulated data and obtained the corresponding pattern. In a last step, we located the topography using eLORETA. As modeled sources were also located in the visual cortex, we are certain that despite the individual electrode montage, source-modeling was performed correctly during this study (Please see Fig. S2 in the supplementary section for a depiction of the simulated pattern and its source reconstruction).

We provide novel evidence, that motor control during BpS is achieved through distinct communication profiles between cortex and muscles in beta and gamma frequency ranges. Considering our findings as well as previous research, it seems that for BpS, CMC and PDC between cortex and muscles are modulated between different movement periods. While we were unable to find differences in CMC between muscles, we were able to show CMC to be elevated during dynamic movement periods (ECC and CON) compared to static ISO. In extension to these observations, PDC also changed most prominently between dynamic movement periods, while remaining mostly stable during ISO. One of the primary aims of motor control research lies in uncovering central-nervous strategies that collectively enable uniquely fine-tuned, task-specific muscle actions. Our study serves as an important step toward a better understanding of this relationship between cortex and muscles during multi joint compound movements.

## Materials and Methods

### Participants

11 healthy, male participants (age: 27.9 ± 5.1 years (mean ± SD)) were enrolled in the present study. The study was approved by the local ethics committee of the Medical Faculty at the University of Leipzig (ref.-nr. 466/17-ek) and all participants gave their written informed consent to participate in the experiments in accordance with the Declaration of Helsinki. Participants were excluded from the present study in case the following exclusion criteria were present: history of neurological or psychiatric disease, intake of centrally acting drugs, caffeine or alcohol intake within 24 hours before the experiment, acute, chronic and/or inadequately regenerated pathologies of the knee joint, the ankle joints and/or the spine (to minimize the risk of injury). Also, participants with regular practice of sports (>3 h./week) were excluded from participation in this study. This was motivated by the fact that previous studies have shown sports expertise to influence CMC occurrence which would affect respective analyses^42^.

### Procedure

**Figure 5:**
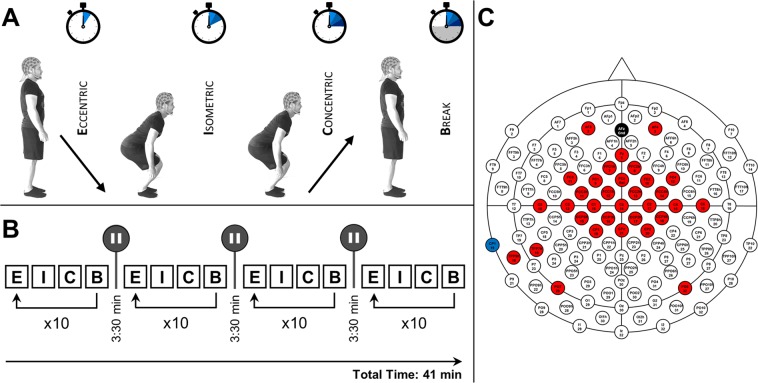
Study design and EEG configuration. (**A**) Depicts a subdivision of the squat movement. As visualized, each squat repetition is divided into three movement periods (ECC, ISO, and CON), with each period having a duration of 5 seconds. After every concentric period, a repetition break period of 30 seconds follows. **(B)** Displays an overview of the study-session timeline. Squats are performed during “activity blocks” of ten repetitions being performed in the manner described above. Each “activity block” is followed by an inter-block break (3:30 min). Additionally, the total session time is illustrated (41 min). Please note that the displayed participant gave written informed consent to use the pictures illustrating the study design. (**C**) Features the individually configured EEG cap used in this study. Areas colored in red implicate utilized EEG channels. Black and blue areas indicate ground and reference electrode positions, respectively.

Each participant completed 40 trials of the squat task. Trials were performed as blocks of 10 repetitions, with each block being separated by breaks of 3:30 min to avoid cumulative effects of peripheral fatigue. Each squatting repetition was divided into three movement periods (ECC – muscles are elongated during contractions; ISO – muscles maintain length during contractions and CON – muscles shorten during contraction), making up three conditions in total. Each period had a duration of 5 seconds. After completion of one repetition (ECC - ISO - CON), an inter-repetition break period (30 seconds) followed on. All period initiations were visually cued on a standard PC monitor running Presentation 16.5 software (NeuroBehavioral Systems, Albany, NY, USA). All participants were naïve for the task (BpS). For an illustrated overview of our study design, please see Fig. 5.

### Behavioral task (BpS)

At the beginning of each experimental session, instructions were given, focusing on the correct execution of BpS. Participants were instructed to plant their feet and exert force without raising their heels during the performance of BpS. Additionally, each participant was instructed to keep a slight lumbar lordosis during BpS. During the entire BpS, all participants had to let their arms hang down and were instructed to keep their heads aligned with their spine. Initial instructions were followed by a brief (3 min) warm-up program comprising supervised executions of dynamic squats without additional weight and focusing on the aforementioned key aspects of correct movement execution, i.e. A, planting of the feet and B, slight lumbar lordosis. For each repetition, participants were instructed to squat down until a knee angle of 95° degrees was reached (squatting depth was established by way of a protractor), meaning that participants started out with the legs completely extended at onset of eccentric movement periods, squatted down until an angle of 95° was reached, maintained this position during the isometric period and extended their legs again during the concentric movement period (cf. Fig. 5).

### EMG recordings

For EMG recordings, a wireless Desktop Transmission System (NORAXON Inc., Scottsdale, AZ, USA) was employed. EMG signals from four bilateral muscles mainly active during squat execution were measured in this study. Bipolar surface electrodes (Ag/AgCl; diameter: 1 cm) were mounted bilaterally on four muscles: *M. vastus lateralis* (VLr & VLl), *M. vastus medialis* (VMr & VMl), *M. tibialis anterior* (TAr & TAl), *M. erector spinae* (ESr, ESl) in accordance to SENIAM electrode position recommendations^69^. Inter-electrode distance was standardized through fixed electrode diameters at 2 cm. The skin of each participant was prepared by removing hair (by means of shaving) around the electrode area, as well as exfoliating the skin. Double-sided tape was used to fixate all transmitters, which were placed in vicinity of the electrodes. Additionally, electrodes were placed in a parallel orientation relative to the muscle fibers. For purposes of synchronizing movement onsets, the display of each movement period onset was synchronously triggered on a PC screen. In summary, data were recorded from 8 channels with a sampling frequency of 3000 Hz, an input impedance >100 MΩ, Common Mode Rejection Ratio (CMRR) > 100 dB, a gain of 500 and a low-pass filter of 500 Hz (Please see Fig. S3 in the supplementary section for an overview of raw EMG data for all recorded muscles, respectively).

### EEG recordings

EEG data were recorded using a wireless 32-channel EEG system (LiveAmp, Brain Products GmbH, Gilching, Germany) with an active electrode setup (actiCAP, Brain Products GmBH). For the purpose of densely covering bilateral sensorimotor areas, 32 electrodes were individually placed on a 128-channel-electrode-cap (cf. Fig. 5C). Ground and reference electrodes were placed on Fpz and left mastoid, respectively. The impedance of all electrodes was kept below 15 kΩ throughout the experiment. Data was transmitted wirelessly to a working station via a Bluetooth transmitter included in the LiveAmp module. Conductive gel (SuperVisc High-Viscosity electrolyte gel) was used to establish contact between the skin and the electrodes and to lower impedance. In summary, EEG data were recorded from 32 channels with a sampling frequency of 500 Hz, an input impedance >100 MΩ and a Common Mode Rejection Ratio (CMRR) > 80 dB. During recording, a band-pass filter between 1–100 Hz was used. Synchronous recordings of both EEG and EMG were ensured through a trigger device (NORAXON Inc., Scottsdale, AZ, USA) that sent out triggers to both recording systems simultaneously.

### EMG and EEG processing

Processing of EMG and EEG signals was carried out using custom software and the BBCI toolbox^70^. EMG data were first decimated (data were low-pass filtered at 200 Hz prior to downsampling) to 500 Hz to match EEG sampling frequencies and subsequently high-pass filtered at 10 Hz, motivated by the fact that the power density function of surface EMG signals has insignificant contributions at frequencies <10 Hz^71^. Data was first divided relating to the respective conditions (ECC, ISO, CON), resulting in 40 5-sec trials per condition. As TA is contracting concentrically during ECC and eccentrically during CON, TA ECC and TA CON were flipped for all analyses. EMG data were subsequently full wave rectified. Power spectral densities (PSD) were estimated using Welch’s method.

EEG data were first filtered by a narrow band stop (notch) filter with a center frequency of 50 Hz and 2 Hz bandwidth to exclude artifacts due to 50 Hz mains. Additionally, EEG data were bandpass filtered at a passband of 0.5–100 Hz. Subsequently, all EEG signals were cleaned to remove eye-blink and scalp EMG artifacts by means of independent component analysis (ICA), using FastICA algorithms^72,73^ implemented in the BBCI toolbox^70^. On average, 2.5 ± 1.2 components were excluded. All filtered signals and their respective components were (A) visually inspected and (B) automatically labeled for artifact rejection through Multiple Artifact Rejection Algorithm (MARA), which is an algorithm trained using selected expert ratings^74,75^. Consequently, it can detect eye-, muscle-, and loose electrode artifacts. All components containing artifacts were excluded from the EEG signals.

### Regression-CMC analysis (r-CMC)

In the present study, we used regression CMC (r-CMC), a multivariate method introduced by Bayraktaroglu, *et al*.^8^, which has been used in a number of previous studies^8,76,77^. This method finds spatial filters for EEG maximizing the synchronization between EEG and EMG activity at a specific frequency range simultaneously using information from all available EEG channels. In a first step, dimension reduction of EEG data was achieved via Principal Component Analysis (PCA), where PCA components are selected, so that they accounted for 99% of the variance in EEG data in a frequency range of interest. This is an important step to avoid collinearity between multi-channel EEG data (serving as predictors). Secondly, a multiple regression was carried out, which utilizes the obtained PCA components as predictors for the EMG signal to find linear combinations of all EEG input variables maximally explaining channel-wise EMG activity. Coherence was then estimated between projected EEG components and EMG signals. For further details refer to Bayraktaroglu *et al*. 2011. All data were split per period and into 500-ms segments (400 in total) which were windowed with a Hanning window and had no overlap (resulting in a frequency resolution of 2 Hz). To evaluate the significance of r-CMC results, permutation testing was carried out, where EMG segments were shuffled with respect to the corresponding EEG data, resulting in the distribution of r-CMC values from 500 permutations. Lastly, results were deemed significant, when unshuffled coherence values were higher than the 95th percentile of shuffled data. To uncover differences in CMC, coherence estimates were summed across three frequency bands of interest: (1) *Beta* (13–30 Hz), (2) *Gamma* (30–44 Hz) and (3) *High Gamma* (44–60 Hz). We chose to analyze CMC as areas of coherence, i.e. summed CMC estimates (${{CMC}}_{{area}}$) over specific frequency bands rather than peak coherence, as ${{CMC}}_{{area}}$ appears to be the most physiologically relevant measure concerning this matter^37,42,78^. For this purpose, significant CMC estimates were summed within each frequency band to yield band-specific ${{CMC}}_{{area}}$^78,79^. ${{CMC}}_{{area}}$ were then pooled for muscles and movement periods. Normal distribution of all variables was assessed through Lilliefors-testing (α = 0.05). Two-way repeated measures ANOVA (rmANOVA) were conducted to determine frequency band related differences in ${{CMC}}_{{area}}$ between muscles and movement periods, with post-hoc Bonferroni tests and simple effect tests being carried out when appropriate. To avoid skewness and normalize variance, all data were log-transformed prior to statistical analyses^78,79^. For all statistical comparisons, a p-value of p < 0.05 was considered significant. All p-values adjusted for multiple comparisons are reported with the results.

### Source-Localization (eLORETA)

To localize neuronal current sources underlying the obtained scalp topographies, a distributed inverse-modeling approach was chosen. Here, we employed the exact low-resolution electromagnetic tomography (eLORETA) algorithm with the regularization parameter being 0.05, as introduced by Pascual-Marqui^80^, to identify neuronal sources corresponding to the optimized patterns obtained with r-CMC, i.e. EEG patterns maximally explaining each EMG channel, respectively. These sources were then mapped using the New York head model^81,82^ with approximately 2000 voxels. Voxel values of each source inside the inverse model were thresholded at 95% of maximum overall voxel activity to capture most pronounced activity. We used EEGLAB to create scalp plots^83^ and the MATLAB toolbox METH by Guido Nolte (https://www.uke.de/english/departments-institutes/institutes/neurophysiology-and-pathophysiology/research/research-groups/index.html) to illustrate source localization results.

### Partial directed coherence analysis

As statistical dependencies between EEG and EMG activity, outlined through coherence, are undirected, we utilized causal multivariate autoregressive models (MVAR) to obtain partial directed coherence (PDC) as a measure of connectivity from MVAR coefficients^30^. PDC provides an account of Granger causality in the frequency domain making it feasible to approximate directed connectivity^84^. We employed the Extended Multivariate Autoregressive Modelling Toolbox (eMVAR) MATLAB (MathWorks) toolbox^29^ to estimate vector autoregressive (VAR) models of sets of signals, comprising of projected EEG components and EMG signals for all muscles and movement periods obtained during the first two steps of r-CMC analyses (for reference, please see paragraph: *Regression-CMC analysis (r-CMC)* above). All model data were decimated to 125 Hz prior to model fitting to avoid redundancy^85^. Optimal model orders were valued by means of Akaike information criterion (AIC) (maximum model order: 20). PDC was subsequently derived from fitted MVAR model components in both possible DIF (i.e., (1) ${{DIF}}_{{EEG}-{EMG}}$ vs. (2) ${{DIF}}_{{EMG}-{EEG}}$). We used a surrogate data approach to assess significance of PDC data. Causal Fourier Transform algorithm (CFT) was used to estimate surrogate data, as it is has been successfully tested on EEG-based PDC data^86^. 200 permutations were used to build surrogate data distributions, with significance being established when PDC vectors eclipsed the 95^th^ percentile of all surrogate data. Significant PDC estimates were summed within each frequency band to yield band-specific ${{PDC}}_{{area}}$. ${{PDC}}_{{area}}$ were then pooled for muscles and movement periods. To obtain results regarding dominant DIF between EEG and EMG data, we compared significant, frequency band summed PDC estimates (${{PDC}}_{{area}}$) in both directions (i.e., (1) ${{DIF}}_{{EEG}-{EMG}}$ vs. (2) ${{DIF}}_{{EMG}-{EEG}}$) with higher magnitudes reflecting dominant DIF. All results were subsequently pooled per frequency band, muscle, movement period and DIF across all participants. Three-way repeated measures ANOVA (rmANOVA) were conducted to determine frequency band related differences in ${{PDC}}_{{area}}$ between muscles, movement periods and DIF, with post-hoc Bonferroni tests and simple effect tests being carried out when appropriate. To avoid skewness and normalize variance, all data were log-transformed prior to statistical analyses. For all statistical comparisons, a p-value of p < 0.05 was considered significant. All p-values adjusted for multiple comparisons are reported with the results.

## Supplementary information

Supplementary Information.

Supplementary Information2.

## Data Availability

The data that support the findings of this study are available on request from the corresponding author, R.K. The data are not publicly available due to data protection policies practiced at our institute (Max Planck Institute for cognitive and brain sciences in Leipzig), e.g. their containing information that could compromise the privacy of research participants.
