# Supplementary material for: Corticomuscular interactions during different movement periods in a multi-joint compound movement
Source: Sci Rep. 2020 Mar 19;10:5021. doi: 10.1038/s41598-020-61909-z (PMC7081206; doi:10.1038/s41598-020-61909-z)

Corticomuscular interactions during different movement periods in a multi-joint compound movement

Rouven Kenville[1,2], Tom Maudrich[1,2], Carmen Vidaurre[5,6], Dennis Maudrich[2], Arno Villringer[2,7,8], Vadim V. Nikulin[2,3,4*§], Patrick Ragert[1,2§]

[1]Institute for General Kinesiology and Exercise Science, Faculty of Sports Science, University of Leipzig, D-04109 Leipzig, Germany

[2]Max Planck Institute for Human Cognitive and Brain Sciences, Department of Neurology, D-04103 Leipzig, Germany

[3]Centre for Cognition and Decision Making, National Research University Higher School of Economics, Moscow, 101000, Russian Federation

[4]Neurophysics Group, Department of Neurology, Charité-University Medicine Berlin, Campus Benjamin Franklin, Berlin, 10117, Germany

[5]Dpt. of Statistics, Informatics and Mathematics, Public University of Navarre, Pamplona 31006, Spain

[6]Machine Learning Group, Faculty of EE and Computer Science, TU Berlin, Berlin 10587, Germany

[7]MindBrainBody Institute at Berlin School of Mind and Brain, Charité-Universitätsmedizin Berlin and Humboldt-Universität zu Berlin, 10099, Germany

[8]Clinic for Cognitive Neurology, University Hospital Leipzig, D-04103 Leipzig, Germany

§ contributed equally

*Correspondence:

Rouven Kenville, Max Planck Institute for Human Cognitive and Brain Sciences, Department of Neurology, D-04103 Leipzig, Germany, Phone: +49 341 9940-2407, Email: kenville@cbs.mpg.de

Supplementary section

Figure S1 - CMC spectra overview. Illustrated are grand-averaged CMC spectra with individual CMC spectra indicated through transparent lines. Only CMC values deemed significant after permutation are illustrated. Columns represent movement periods: ECC (blue), ISO (red) and CON (gray), rows indicate muscles.

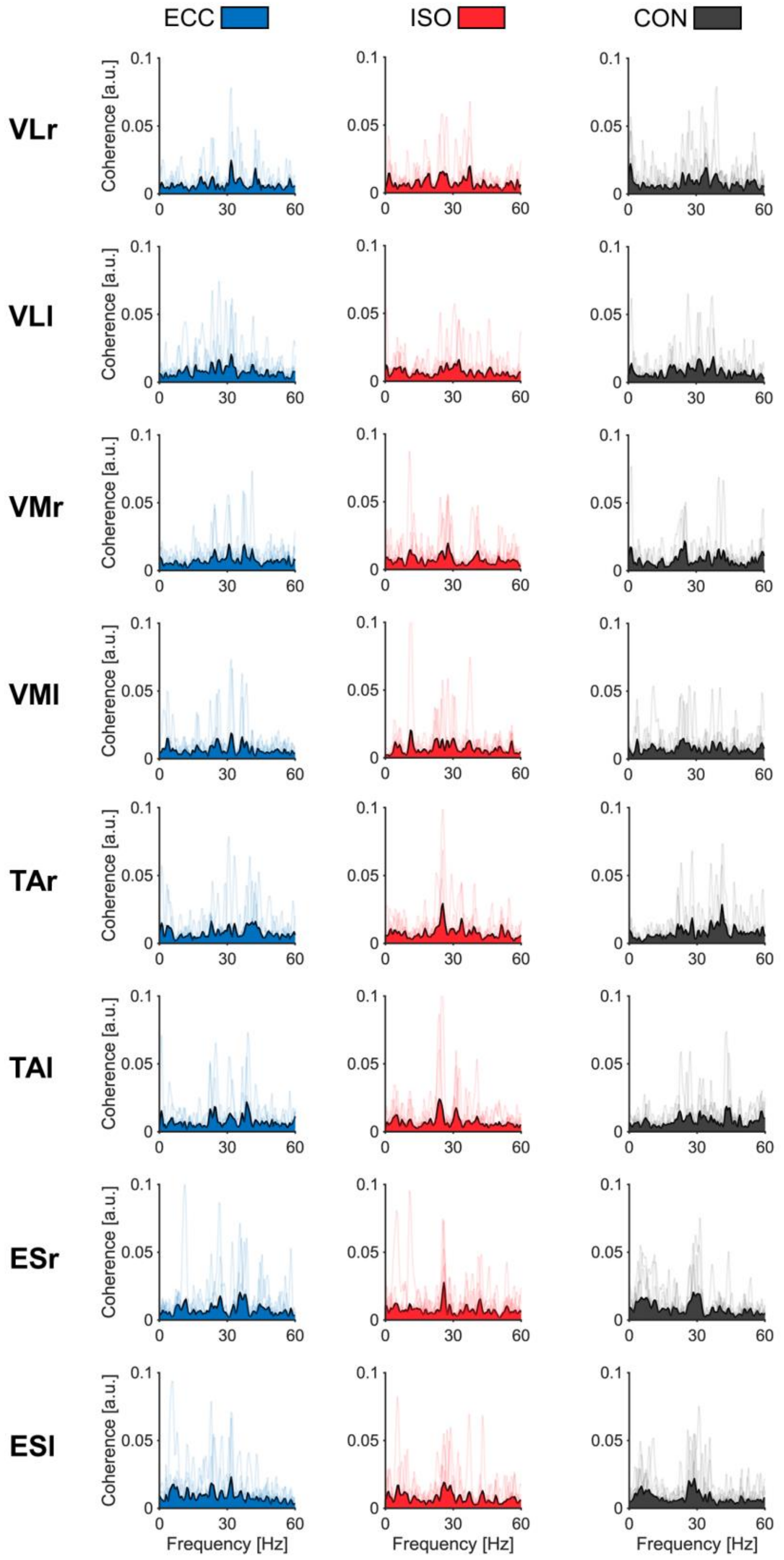

Figure S2 - Source - localization of simulated data. **(A)** Scalp topography and **(B)** corresponding source-localization results of simulated EEG sources located in the visual cortex coupled with an EMG source. Electrode positions correspond to those used during all measurements. We used EEGLAB [83] to create scalp plots in section **(A)** and the MATLAB toolbox METH by Guido Nolte (https://www.uke.de/english/departments-institutes/institutes/neurophysiology-and-pathophysiology/research/research-groups/index.html) to illustrate source localization results in section **(B).**

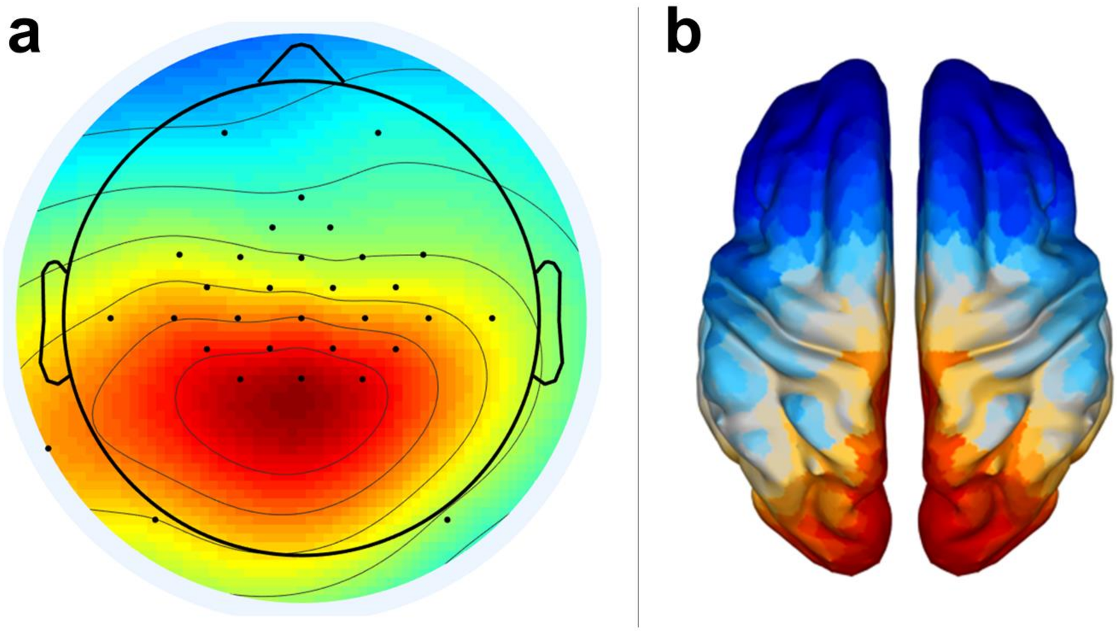

Figure S3 - Overview of EMG activity per muscle and period. EMG bursts (40 per period) were averaged across muscles, epochs and participants. Each column represents different movement periods: ECC (blue), ISO (red) and CON (gray). Each row represents distinct muscles with labels next to each burst. Please note different scaling between muscles.

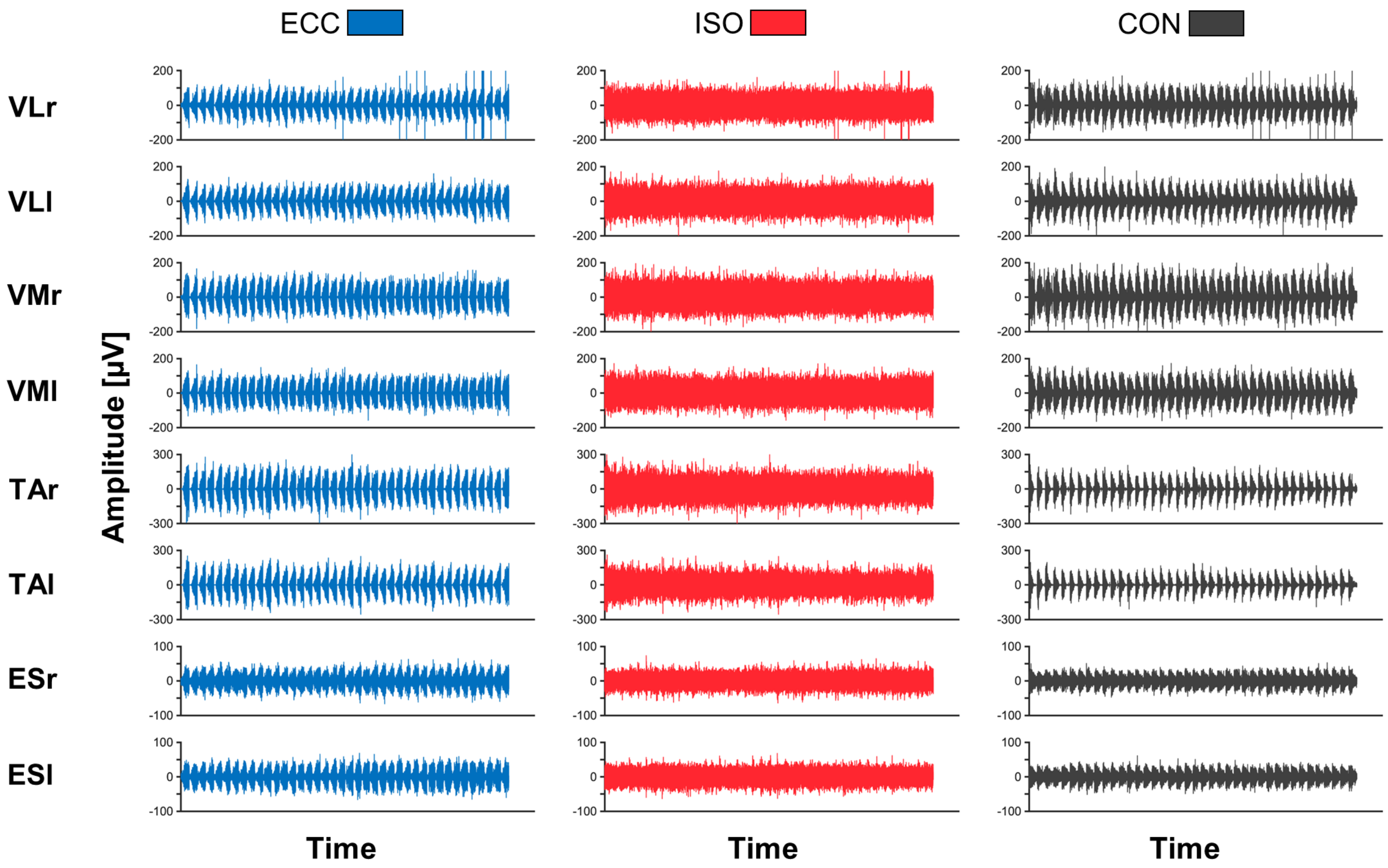

Supplement: Supplementary file 2 — Supplementary Information2. [file 41598_2020_61909_MOESM2_ESM.pdf]
